# Pembrolizumab and reactivation of TB: is there a link?

**DOI:** 10.5588/ijtldopen.26.0341

**Published:** 2026-10-12

**Authors:** N. Chaturvedi, P. SONI, I.N. Shaikh, S. Singhania

**Affiliations:** 1 Department of General Medicine, Kokilaben Dhirubhai Ambani Hospital, Mumbai, India;; 2 Department of Pulmonary Medicine, Kokilaben Dhirubhai Ambani Hospital, Mumbai, India;; 3 Department of Medical Oncology, Kokilaben Dhirubhai Ambani Hospital, Mumbai, India.

**Keywords:** Immunotherapy, malignancy, opportunistic infection

Immune checkpoint inhibitors (ICIs), particularly programmed cell death protein 1 (PD-1) inhibitors such as pembrolizumab, have transformed cancer treatment by enhancing anti-tumour immunity. However, immune activation may also disrupt TB infection (TBI) control, leading to reactivation, especially in TB-endemic regions. The immunopathology of TB reactivation is a complex phenomenon. Diagnosis is challenging because TB can mimic disease progression or immune-related inflammation on imaging. We present three cases of TB occurring during pembrolizumab therapy, highlighting diagnostic dilemmas and the importance of early microbiological evaluation and prompt anti-tubercular therapy (ATT) – see Table.

**Table.** Clinical timeline and characteristics of TB developing during pembrolizumab therapy.

Variable	Case 1	Case 2	Case 3
Age/gender	76-year-old man	56-year-old man	61-year-old man
Co-morbidities	Hypertension	Diabetes mellitus	None
Prior TB history/contact	None	None	None
Underlying malignancy	Clear cell renal cell carcinoma (pT3c)	Metastatic squamous cell carcinoma of pyriform fossa	Metastatic gallbladder carcinoma
Treatment regimen	Pembrolizumab	Paclitaxel + carboplatin + pembrolizumab	Gemcitabine + cisplatin + pembrolizumab
Number of pembrolizumab cycles before TB diagnosis	12 cycles	3 cycles	4 cycles
Duration of immunotherapy before TB diagnosis	9 months	2 months	3 months

## CASE 1

A 76-year-old man with a known history of hypertension presented with left flank pain, flank fullness, and significant weight loss of 7 kg over 3 months. He had no past history of TB or TB contact exposure. He was diagnosed with clear cell renal cell carcinoma (pT3c) and subsequently underwent radical nephrectomy, followed by adjuvant therapy with pembrolizumab. After completing eight cycles over 6 months, positron emission tomography combined with computed tomography (PET–CT) revealed pleural thickening, pleural effusion, and mediastinal lymphadenopathy, which were initially interpreted as disease progression, and hence pembrolizumab was continued (Figure). After receiving a total of 12 cycles of pembrolizumab over 9 months, imaging demonstrated improvement in pleural disease, but new lung nodules appeared. Subsequently, the patient developed a fever, weight loss, an acute confusional state, and respiratory compromise. High-resolution CT chest revealed cavitatory consolidation and multiple pulmonary nodules suggestive of TB. Bronchoalveolar lavage was 3+ positive for acid-fast bacilli and GeneXpert MTB, confirming pulmonary TB. ATT was initiated, resulting in rapid clinical improvement.

**Figure.**
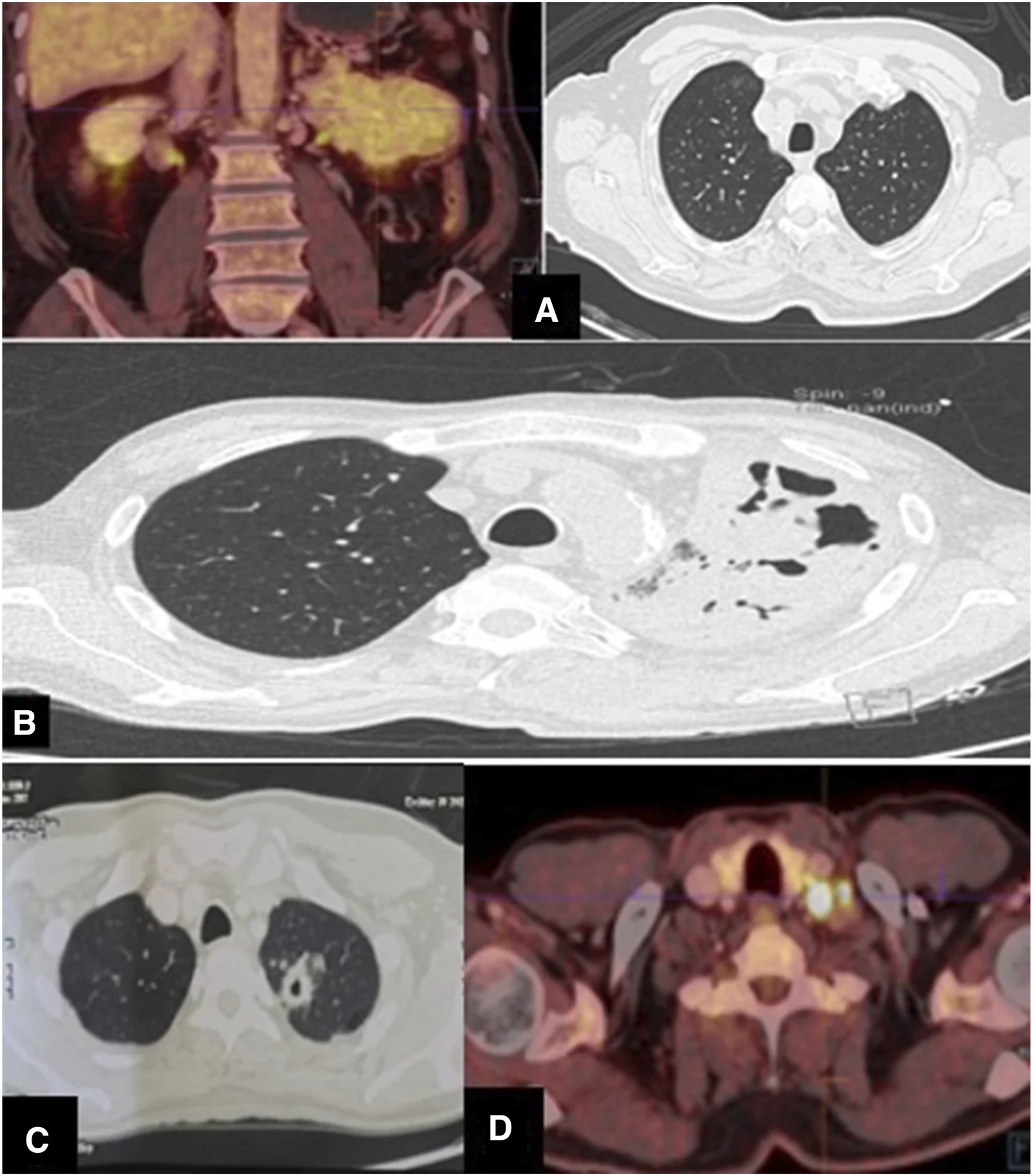
**A:** Case 1, left image: PET–CT showing metabolically active mass lesion involving left kidney suggestive of renal cell carcinoma, right image: normal lung parenchyma; **B:** Case 1, HRCT chest (post-immunotherapy) showing cavitatory consolidation involving the left upper lobe, raising suspicion of TB; **C:** Case 2, HRCT chest showing a cavitatory lesion in the left upper lobe; **D:** Case 3, PET–CT showing metabolically active left cervical lymph node. PET–CT = positron emission tomography combined with computed tomography; HRCT = high-resolution CT.

## CASE 2

A 56-year-old diabetic gentleman came with complaints of progressive dysphagia to solids for 2 weeks along with a change in quality of voice, associated with fever and significant weight loss. He had no past history of TB or TB contact exposure. After extensive evaluation, he was diagnosed with metastatic squamous cell carcinoma of the pyriform fossa and was started on paclitaxel, carboplatin, and pembrolizumab. After receiving three cycles over 2 months, he developed a fever, cough, and breathlessness. Imaging showed new left upper lobe consolidation, and sputum was 3+ positive for acid-fast bacilli, confirming pulmonary TB (Figure). The patient improved on ATT. Follow-up PET–CT showed improvement of the cavitatory lung lesion, suggesting response to ATT.

## CASE 3

A 61-year-old man with no known co-morbidities presented with complaints of loss of appetite, abdominal pain, generalised weakness for one month, intermittent fever, and significant weight loss. He had no past history of TB or TB contact exposure. After evaluation, he was diagnosed with metastatic gallbladder carcinoma. He was started on and treated with gemcitabine, cisplatin, and pembrolizumab. A follow-up PET–CT after four cycles over 3 months showed an excellent response of the primary malignancy but newly appearing fluorodeoxyglucose-avid left cervical lymph nodes, raising concern for metastatic disease (Figure). A lymph node biopsy revealed caseating granulomatous inflammation and was also positive for GeneXpert MTB with a cycle threshold value of 30, confirming *Mycobacterium tuberculosis*. ATT was started successfully, and the patient subsequently underwent radical cholecystectomy with good postoperative recovery.

There has been a huge influx of immunotherapy molecules in the treatment of various cancers. The results of these therapies have been very encouraging, but they come with their unique basket of possible complications, most occurring as a result of direct drug toxicity to a variety of organs, especially lungs, due to disrupted immune regulation. Although ICIs are designed to enhance anti-tumour immunity, growing evidence suggests that disruption of immune regulatory pathways may paradoxically predispose susceptible individuals to uncontrolled inflammatory responses and possibly breakdown of TBI containment. The above cases highlight the emerging association between ICIs, particularly PD-1/PD-L1 inhibitors such as pembrolizumab, and reactivation of TBI.

Under normal conditions, TBI exists in a state of immunological equilibrium in which mycobacteria are contained within granulomas by coordinated macrophage and T-cell activity. CD4^+^ and CD8^+^ T lymphocytes produce regulated levels of interferon-gamma (IFN-γ) and tumour necrosis factor-alpha (TNF-α), while PD-1 signalling limits excessive immune activation and prevents immune exhaustion. Within TB granulomas, PD-1 and PD-L1 are expressed on T cells and macrophages, respectively, indicating that this checkpoint pathway plays an important immune regulatory role in maintaining granuloma integrity.^1^ Blockade of PD-1/PD-L1 signalling by ICIs leads to enhanced Th1 activation, increased production of IFN-γ, TNF-α, and IL-2, and amplified cytotoxic T-cell responses. Although theoretically this could improve antimycobacterial immunity, excessive cytokine activation instead appears to promote granuloma destabilisation and disorganised inflammatory infiltration. Experimental studies have demonstrated that PD-1 knockout mice infected with *M. tuberculosis* develop severe necrotising inflammation, higher bacterial burdens, and rapid mortality, suggesting that PD-1 signalling is protective against TB-related immunopathology and its inhibition may trigger reactivation of latent infection rather than improved microbial clearance.^2–4^ Ratner et al.^5^ and Cantini et al.^6^ have further emphasised that TB associated with ICIs may represent immune dysregulation rather than primary infection alone and that these cases often require careful interpretation of clinical, radiological, and microbiological data. Previous reports have proposed an IRIS-like phenomenon secondary to PD-1/PD-L1 inhibition as a mechanism for TB development.^7^ However, our cases demonstrated a high bacillary burden of 3+ AFB-positive in two of the three patients, suggesting that an IRIS-like reaction alone cannot fully explain the pathophysiology of TB in these cases, and our findings are consistent with the findings in the PD-1 knockout mice model. ICIs also commonly cause immune-related adverse reactions, including colitis, hepatitis, and pneumonitis. Excess cytokine production like TNF-α has been implicated as one of the reasons for this, and infliximab has been used as an antidote. This again appears consistent with the fact that immune activation, including excess TNF-α activity with pembrolizumab therapy, does not appear to offer protection against TB.

Clearly, there have been other risk factors in all three cases for reactivation of TB, which include active malignancy, TB prevalence in the community, and diabetes. Cancer itself further contributes to susceptibility, particularly during the first year following diagnosis and initiation of therapy, when immunological dysregulation is greatest.^8^ TB reactivation associated with checkpoint inhibitors has been observed to typically develop within weeks to months after initiation of therapy, as seen in our cases. Importantly, these presentations may initially mimic tumour progression or immune-related pneumonitis, thereby delaying diagnosis and appropriate therapy. A systematic review by Zaemes et al.^7^ identified 16 cases of active TB developing during ICI therapy, with most cases occurring in patients receiving PD-L1 inhibitors at a median of 6.3 months after treatment initiation. The review highlights that TB reactivation can involve both pulmonary and extra-pulmonary sites and may lead to fatal complications, suggesting the need for TB screening and close monitoring in high-risk patients receiving pembrolizumab or other ICIs. These observations underscore the importance of risk stratification and screening for TBI before initiation of immunotherapy. Several authors have proposed routine IFN-γ release assay testing prior to anti-PD-1 therapy, especially in patients with additional risk factors.^9^ Although evidence specific to checkpoint inhibitors remains limited.

In conclusion, pembrolizumab and other ICIs may contribute to reactivation of TBI through disruption of granuloma immune regulation. The immune reactivation induced by pembrolizumab does not offer protection against TB, and reactivation cannot be considered as part of IRIS. TB occurring after immunotherapy may mimic malignancy progression or immune-mediated toxicity, making early recognition clinically challenging. While pulmonary TB is the most common presentation, extra-pulmonary manifestations have also been reported. Careful risk stratification and TBI is screening should be considered before initiation of immunotherapy, particularly in high-risk individuals and TB-endemic settings.
